# Genometa - A Fast and Accurate Classifier for Short Metagenomic Shotgun Reads

**DOI:** 10.1371/journal.pone.0041224

**Published:** 2012-08-21

**Authors:** Colin F. Davenport, Jens Neugebauer, Nils Beckmann, Benedikt Friedrich, Burim Kameri, Svea Kokott, Malte Paetow, Björn Siekmann, Matthias Wieding-Drewes, Markus Wienhöfer, Stefan Wolf, Burkhard Tümmler, Volker Ahlers, Frauke Sprengel

**Affiliations:** 1 Pediatric Pneumology, Allergology and Neonatology, Hannover Medical School, Hannover, Lower Saxony, Germany; 2 Department of Computer Science, University of Applied Sciences and Arts, Hannover, Hannover, Lower Saxony, Germany; University College Dublin, Ireland

## Abstract

**Summary:**

Metagenomic studies use high-throughput sequence data to investigate microbial communities *in situ*. However, considerable challenges remain in the analysis of these data, particularly with regard to speed and reliable analysis of microbial species as opposed to higher level taxa such as phyla. We here present Genometa, a computationally undemanding graphical user interface program that enables identification of bacterial species and gene content from datasets generated by inexpensive high-throughput short read sequencing technologies. Our approach was first verified on two simulated metagenomic short read datasets, detecting 100% and 94% of the bacterial species included with few false positives or false negatives. Subsequent comparative benchmarking analysis against three popular metagenomic algorithms on an Illumina human gut dataset revealed Genometa to attribute the most reads to bacteria at species level (i.e. including all strains of that species) and demonstrate similar or better accuracy than the other programs. Lastly, speed was demonstrated to be many times that of BLAST due to the use of modern short read aligners. Our method is highly accurate if bacteria in the sample are represented by genomes in the reference sequence but cannot find species absent from the reference. This method is one of the most user-friendly and resource efficient approaches and is thus feasible for rapidly analysing millions of short reads on a personal computer.

**Availability:**

The Genometa program, a step by step tutorial and Java source code are freely available from http://genomics1.mh-hannover.de/genometa/ and on http://code.google.com/p/genometa/. This program has been tested on Ubuntu Linux and Windows XP/7.

## Introduction

Metagenomics, the analysis of microbial communities directly within their natural environments, continues to gain traction in both the environment and in the clinic. In metagenomics, sequence reads can be used to predict both the abundance and functional capacity of the microbes present by molecular means. Sequence read data from high throughput sequencing platforms like Illumina and SOLiD are by far the most cost-effective per base pair sequenced [1], yet downstream analysis remains challenging, with algorithmic speed an issue. Despite this, extensive short read datasets are beginning to accumulate [2], [3], [4].

Sequence reads in bacterial metagenomic analyses can be derived by whole genome shotgun sequencing, or targeted sequencing of 16S rRNA amplicons. These alternative techniques do lead to significant taxonomic differences in results, based upon the evaluation of 33 metagenomes [5]. In other words, the decision to select targeted 16S amplicon sequencing or untargeted whole genome sequencing will lead to different predictions of the taxonomy of a metagenome. Sequencing of 16S rRNA remains a popular approach [6] in metagenomics despite its well known limitations [7], [8]. Estimates of taxon abundances can be biased by large differences in rRNA copy number between even closely related species [9], and the fact that not all rRNA genes amplify with PCR primers [10]. In fact, the number of copies of the rRNA gene in bacteria range from 1–15 [9], rendering rRNA-based approaches more suitable for qualitative than quantitative metagenomics. Because of these reasons, we anticipate whole genome shotgun metagenomes will be preferable to sequencing of rRNA amplicons in the future.

Ideally, researchers require programs which can perfectly assign reads to individual microbial strains. This goal is not realistically possible due to the very high sequence similarity between strains, the reads errors inherent to sequencing, and the lack of reference genome sequences for some phyla. However, the optimal result for a metagenome dataset must remain species level read assignments, and not unspecific matches to phyla such as Firmicutes or Proteobacteria. Attributions to higher taxonomic levels, while indicative of the presence of unsequenced phyla in the metagenomic sample, are not a satisfactory solution because taxonomy becomes increasingly arbitrary with distance from the species unit [11]. Hence, algorithms which can generate more precise taxonomic assignments are necessary.

A key strength of using reads from the whole genome is the ability to probe the gene content of the organisms present. With this method predictions can be made to establish the pangenome of the community under investigation, and even discover new genes. For example, Hess and coworkers [4] found over 27,000 putative genes putatively involved in carbohydrate metabolism and could assemble 15 uncultured microbial genomes from a cow rumen metagenome dataset, and could even express novel proteins active against cellulose substrates. Qin and colleagues [3] discovered a core microbial gene set around 150 times larger than the human gene complement after sequencing the microbiomes of 124 European individuals.

As monitoring approaches and reference sequences continue to expand, algorithms which are able to rapidly detect species in large metagenomic datasets will become essential. Furthermore, quick assessment of large datasets is important to facilitate rapid detection of pathogens in the clinic. Existing algorithms of the same speed as the BLAST algorithm are not sufficiently scalable to large datasets [12], and webservers typically provide restrictive limitations or allow others access to sensitive data [13].

We here present Genometa, a robust, fast and accurate system for assignment of short reads from prokaryotic metagenomes which can be run as a Java application on a personal computer or via our webserver. This system allows rapid analysis of the vast datasets generated by next generation sequencers and thus facilitates investigation of complex microbial communities at a greater level of detail. We demonstrate the utility of the approach in assessment of taxonomic origin of millions of reads from a human gut dataset and validate predictions on artificial metagenome datasets of known composition. Genometa achieves a high proportion of read assignments, with few false assignments to species not included in the dataset.

## Methods

### Genometa

The program is an extensive modification of the established Integrated Genome Browser (IGB) genome browser [14]. IGB was selected because of its functionality, clear user interface and extensible, well-documented Java source code. The IGB codebase was forked in order to develop Genometa in a separated subversion repository. SAM to BAM conversion is implemented using the Picard Java library (http://picard.sourceforge.net/), and reads from BAM files are counted, mapped to metadata and subsequently displayed in a histogram and in the genome browser. Initially, support for the Bowtie algorithm [15] has been included in the graphical interface and BWA [16] will be included in the future.

### Reference sequences for metagenomics

1190 prokaryotic chromosomes from various sources were concatenated and used to build a metagenomic reference sequence. These include the August 2010 versions of the NCBI RefSeq collection, the Human Microbiome project [17], the Genomic Encyclopedia for Bacteria and Archaea [18], the Metahit programme [3], and the Moore Foundation Marine Microbial Genome Sequencing Project (http://www.moore.org/marine-micro.aspx). The earliest sequenced strain genome from each species was included using an in-house script. The included genomes are listed within the Genometa program itself. Short reads can then be mapped onto this reference in a similar fashion to that routinely used in genome resequencing. This reference sequence was used to derive the results mentioned in this paper. Reference sequences will be updated regularly using complete and draft genomes from these resources as well as novel resources arising in the future. We have designed a Java program named RefSelector, downloadable from the Genometa website, which allows customisation of the reference sequence. Users modify the taxa in the included list, run RefSelector, and receive a new reference sequence containing only those genomes included in the modified list.

### Accuracy on simulated metagenomes

The advantage of using simulated data to assess a program is that the true origin and method of production of the datasets are known, and hence positive predictions can indeed be verified. Because short reads offer the lowest cost per bp and have been underutilised by metagenome researchers to date, 50 bp (starting from the first base) of sequence were filtered from metagenomic projects with longer reads using an in-house PERL script. As an exception, short reads were extracted from Sanger reads at position 100 to avoid ambiguous bases present at read starts. Default bowtie settings were used for analyses of the SimLC [19] and simulated ocean metagenome datasets. SimLC is a dataset constructed by other researchers to allow objective assessments of metagenome analysis programs. The simulated ocean metagenome was created with Metasim [20] using 100000 reads from ten marine strains: 17391 reads from *Marinomonas* sp. MWYL1, 15665 from *Shewanella loihica* PV-4, 12473 reads from *Oceanobacillus iheyensis* HTE831, 11890 reads from *Nitrosococcus oceani* ATCC19707, 10509 reads from *Alcanivorax borkumensis* SK2, 9252 reads from *Synechococcus elongatus* PCC6301, 6911 reads from *Halobacterium salinarum* R1, 5946 reads from *Prochlorococcus marinus* CCMP1375, 5619 reads from *Nitrosopumilus maritimus* SCM1 and 4344 reads from Candidatus *Pelagibacter ubique* HTCC1062.

### Alignment speed comparison of BLAST and Bowtie

In order to test the alignment speed of BLAST and Bowtie on the same datasets reads were collected from a human gut study [21], a human stool diarrhea study [22], a vineyard study [2] and a cystic fibrosis lung dataset [23]. Bowtie was run within Genometa using default settings with the exception of -p (number of threads) being raised to achieve optimal resource usage. BLAST v2.2.13 was run with the following settings: (blastall -p blastn -d allSpecies_august2010.fa -i $input -e 1e-10 -a 7 -b 30 -v 30 -F F -o $output1). Analyses were run on the same machine, and results were normalised to ensure comparability. Extrapolation of BLAST results was considered only after 24 hours had elapsed in order to save power.

### Benchmarking algorithms on an Illumina human gut microbiome dataset

The first 100,000 Illumina 100 bp reads were extracted from an Illumina human gut dataset derived from stool samples (SRR042027, Human Microbiome Project, [17]). Only 100,000 reads were taken as this represents the upper limit which could (in late 2011) be submitted to one of the metagenome webservers tested. Only the first reads of each read pair were used to ensure comparability between the programs under test, since most lack functionality to deal with paired end reads. The exact bowtie command used for the Genometa assessment was: (bowtie -t allSpecies_august2010 –sam -p 15 -f gut.fa gut.sam). Megan [24], MG-RAST [25], and Carma3 [26] were all run with default settings. The Blast command for Megan was: (blastall -p blastn -d allSpecies_august2010.fa -i $input -e 1e-10 -a 7 -b 30 -v 30 -F F -o $output1).

## Results

### Genometa

**Figure 1:**
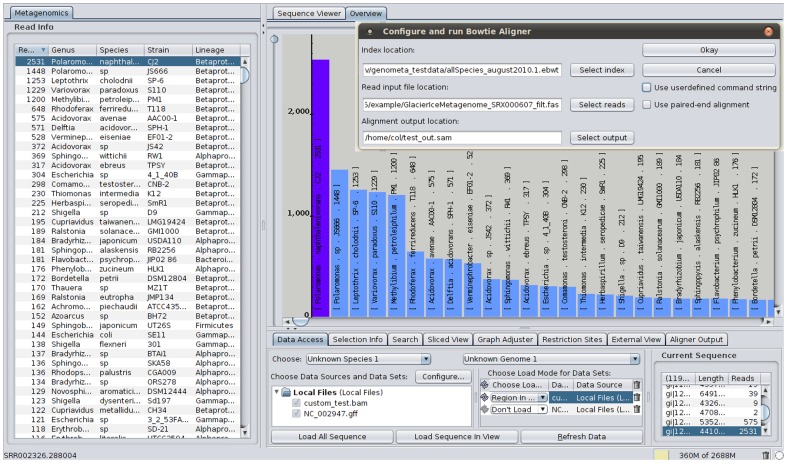
A screenshot displaying key new features with a glacier ice metagenome dataset loaded [**39**]. An aligner can be run with the graphical dialogue (top right) against a reference sequence. Thereafter the resulting file format is converted to the standard BAM format and read in, revealing the number of reads mapped to each species in a sortable list which can be exported for further analysis (left). A bar graph graphically displays the number of reads attributed to each taxon. Clicking on a blue bar takes the user to a genome level view of the distribution of reads mapped against a taxon. Large datasets can thus be easily aligned, analysed and tested for plausibility from a graphical user interface.

The open source genome browser IGB [14] was extended and extensive functionality for a metagenomic scenario was added. New features include integration of SAM/BAM format conversion and reading functionality via the Picard library (http://picard.sourceforge.net/), integration of the Bowtie and bwa aligners [15], [16], summarisation and graphical summaries of results, new visualisations and novel export functions (Figure 1). Bowtie was selected since it is sufficiently fast, accurate and available on all major desktop operating systems. Advanced users can set any Bowtie parameters using a text box, use other alignment programs and read in their own alignments from SAM or BAM files. In other words, Genometa is flexible enough to allow complete access to the changing functions of the integrated aligner. Complex sequence data files can thus be easily created, converted, read in and explored via sorted drop down lists in a user-friendly manner. These features are completely distinct from the functionality that the main IGB developers have been focussing on, such as enhanced performance, improved visualisation tracks, insertions and deletions, plugins and web-based data access. Many of our modifications and improvements will flow back into the main IGB project managed by the Loraine lab (Ann Loraine, pers. comm.).

### Speed comparison to BLAST

Many existing metagenome analysis approaches are based on the BLAST algorithm [27] or others which run at similar speeds [28]. We have previously observed BLAST to be a thorough but slow algorithm for next-generation sequence data, and thus tested the speedup by Bowtie with metagenomic datasets. BLAST was observed to map about 0.25 reads per thread per second in all four datasets (Table 1), whereas Bowtie managed to map between 525 and 1967 reads per thread per second. While the reads from the 454 and Sanger sequenced metagenomes were trimmed to allow alignment by Bowtie, this is a dramatic difference in speed.

**Table 1:** Comparative duration of alignment by BLAST and Bowtie versus the same metagenomic reference for four metagenome datasets.

Dataset	Alignment tool	Number of reads	Alignment duration 7 threads (s)	Normalised alignment duration 1 thread (s)	Reads per thread per second	Dataset reference
Human gut	BLAST	1501409	885898*	6201286	0.24	Kurokawa et al. 2007 [21]
Human gut	Bowtie	1501409	109	763	1967.77	Kurokawa et al. 2007 [21]
Human stool Diarrhea	BLAST	96941	54180	379260	0.26	Nakamura et al. 2008 [22]
Human stool Diarrhea	Bowtie	96941	14	98	989.19	Nakamura et al. 2008 [22]
Vineyard	BLAST	9623513	5854784*	40983488	0.23	Coetzee et al. 2010 [2]
Vineyard	Bowtie	9623513	2617	18319	525.33	Coetzee et al. 2010 [2]
CF lung	BLAST	772097	432374*	3026618	0.26	Willner et al. 2009 [23]
CF lung	Bowtie	772097	69	483	1598.54	Willner et al. 2009 [23]

* duration was extrapolated after ∼24 hours.

### Testing on artificial metagenomes of known composition

**Figure 2:**
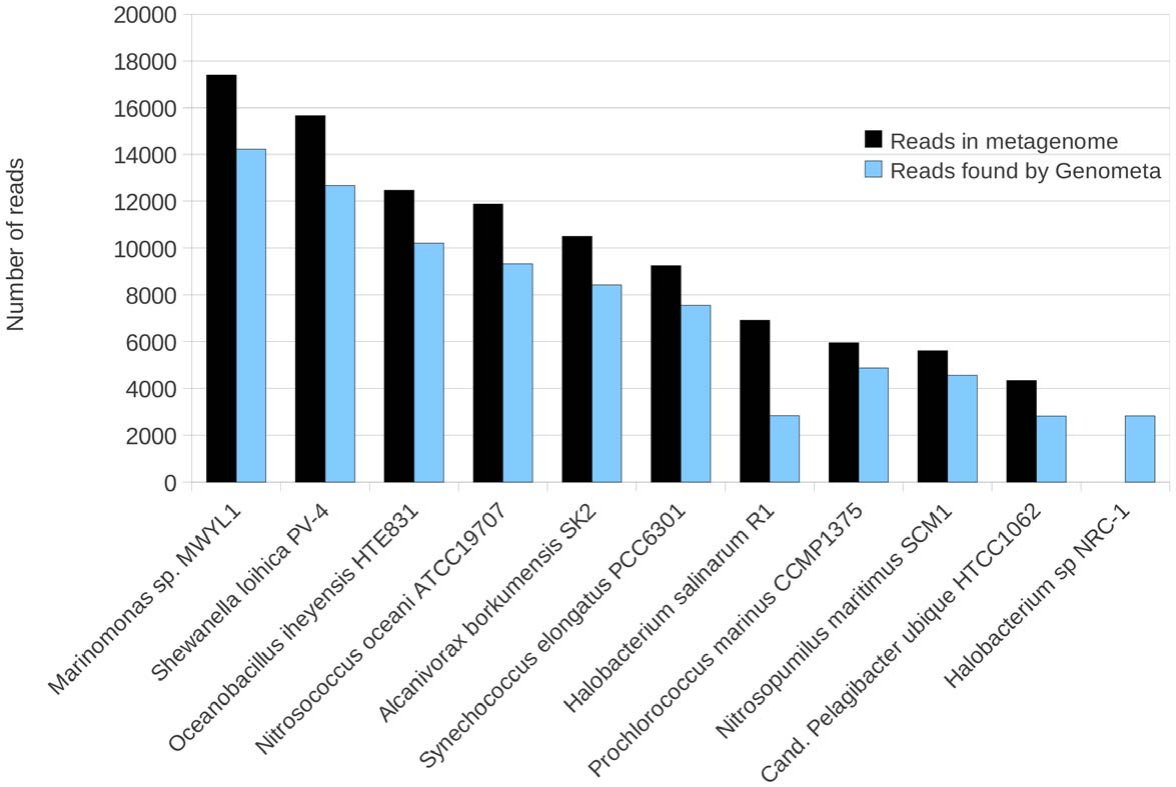
Number of reads per species present in an in-house simulated ocean metagenome compared to the number of reads assigned to a reference containing all known strains by Genometa. All bacterial species present were detected. Reads were retrieved in the same stoichiometric proportions in which they were inserted. *Halobacterium* sp NRC-1 was also detected, but this strain is colinear and practically identical to the included strain *Halobacterium salinarum* R1 [29].

**Figure 3:**
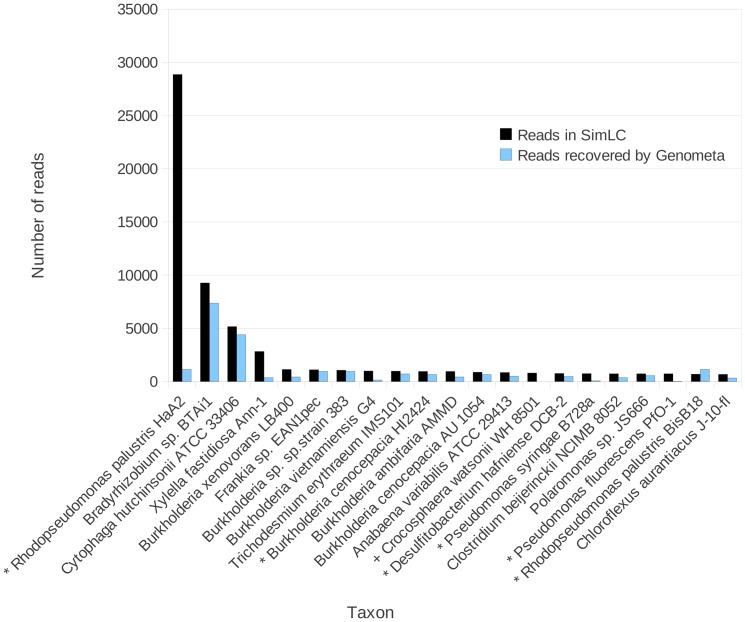
Number of reads from an artifical metagenome of known composition (SimLC dataset; [**19**]) which were included in the metagenome (black bars) and assigned to the correct bacterial species by Genometa (blue bars). Only the top 21 species of the 113 bacteria included in the dataset are shown. Genometa achieves a high accuracy on this dataset. Asterisks indicate strains which are included in the SimLC dataset but not in the Genometa reference sequence. Inter strain differences generally mean less reads are attributed to these taxa. The cross denotes a species which is not present in the Genometa reference sequence.

Genometa was first tested on a composition of simulated, error containing reads derived from ten oceanic bacterial strains. The results clearly reflected the bacteria included in the metagenome, with an average of 75% of reads inserted being correctly assigned to each species (Figure 2). *Halobacterium* sp NRC-1 was also detected, but this strain is colinear and practically identical to the included strain *Halobacterium salinarum* R1 [29]. Because identical strains are included in the reference naturally about half the reads are attributed to each strain. As such this is a taxonomic oversight as to why NRC-1 has not been assigned to the species *H. salinarum* rather than an algorithmic error. Next, a published simulated low complexity dataset comprising 113 strains with introduced simulated errors (SimLC, [19]) was used to validate Genometa performance. A detection threshold of 100 reads was utilised because 100 aligned short reads are sufficient to estimate sequence length and abundance of a taxon, operon or genomic island in a metagenomic dataset (see equations 3–5 in Materials S1). 106 of 113 strains (94%) were detected (Figure 3). Nine species were not detected (false negatives), of which two were not in the reference dataset. For the seven remaining taxa, one was completely undetected, while the genus (but not species) for four taxa was correctly predicted and two species were falsely predicted to be present (false positives).

A number of strains in the SimLC dataset were not included in the Genometa reference, which only includes one strain from each species. The species *Crocosphaera watsonii* WH 8501 and five further strains in Figure 3 were not included (marked by asterisks). Where related strains are not included in the Genometa reference, Bowtie maps some dataset reads to homologous regions of the included strain, but leaves others unmapped. This explains why the strains missing from the Genometa reference, such as *Pseudomonas fluorescens* PfO-1, are present in the results, but have a lower proportion of mapped reads.

On a different note, *R. palustris* BisB18 is assigned more reads than were present from this strain in the SimLC dataset. This can be explained by the presence of reads from four *R. palustris* strains in the entire SimLC dataset. In other words, reads from *R. palustris* strains HaA2, BisB18 and others were only partially assigned to common regions of strain *R. palustris* CGA009, a considerably divergent strain (just 64% query coverage using NCBI Megablast Blast2Seq to strain HaA2) which is included in the Genometa reference. The attributed reads are then presented for both *R. palustris* strains shown, since Genometa only attempts attributions at the species (not strain) level. Where reads from multiple strains are present in the SimLC dataset, summation of the reads would actually be fairer to Genometa, but we prefer to strictly retain the ordering of the SimLC dataset to allow meaningful future comparisons with other metagenome analysers. These examples demonstrate the taxonomic fidelity of short reads, since very few reads from the missing strain are simply mapped to the closest strain.

### Analysis of a human gut metagenome sample

**Figure 4:**
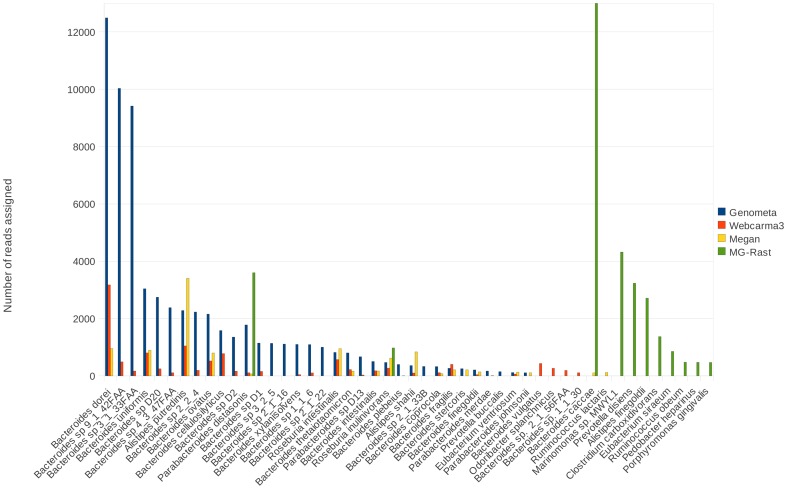
The number of 100,000 Illumina human gut 100 bp reads (SRR042027, Human Microbiome Project, [17]) assigned to bacterial species by four metagenomic programs. Note the general agreement between the different programs but higher number of read assignments achieved by Genometa and MG-RAST. All programs found bacterial species typical of a human gut metagenome.

Finally, a comparative performance analysis of four metagenome analysers on 100,000 100 bp human gut Illumina reads was carried out (Figure 4, SRR042027, Human Microbiome Project, [17]). The leading programs Webcarma3 (webserver), MEGAN (standalone program), and MG-RAST (webserver) were compared against Genometa. Processing times were difficult to measure for the webserver approaches, while Genometa was distinctly faster than the BLASTn analysis used in MEGAN (BLAST: ∼40 hours, Bowtie: ∼1 minute; see also Table 1 for performance on other datasets).

Genometa was able to attribute the most reads (64%). Thereafter followed MG-RAST (62%), while Webcarma3 (11%) and MEGAN (10%) were more conservative. In general, the number of reads attributed by Genometa to these bacterial species correlates with those from Webcarma and MEGAN, although Genometa does place significantly more reads. Two taxa were detected by all four approaches, while twelve taxa were found by three programs. Ten taxa were implicated by Genometa and Webcarma3 only. All programs detected taxa not found by any other, such as *Bacteroides* sp. 3_2_5 (Genometa), *Bacteroides vulgatus* (Webcarma3), *Ruminococcus lactaris* (MEGAN) and *Marinomonas* sp. MWYL1 (MG-RAST). In general, results were as expected for all programs for a human gut dataset, with a high proportion of bacteria from the genera *Bacteroides* and *Alistipes* discovered.

## Discussion

Metagenome sequence analysis has been an area of active research since next-generation sequencers became available [13], [27], [28]. In this study, we introduce a novel standalone metagenomic program specifically designed for the challenges of whole genome short read analysis. We also evaluated the key issues of run time and accuracy against simulated datasets of known composition and an Illumina human gut dataset with benchmarking against three other metagenome analysers.

On the human gut dataset, Genometa and MG-RAST could assign the most 100 bp reads to the listed bacterial species (Figure 4). MEGAN and Webcarma3 were only able to make significantly less assignments, but all approaches predicted a taxonomic composition close to that observed by numerous other human gut studies [3], [21]. While there was considerable overlap between results from the four programs on the human gut dataset, there were also significant differences. These differences are for most part probably due to the difference in reference sequences between Genometa on the one hand and Webcarma and MG-RAST on the other, the latter of which are both webservers and hence cannot be fed the exact same reference sequence. For example, MG-RAST assigned 43215 reads to *Bacteroides caccae*, which was not found by the other programs except for Megan (104 reads). We speculate that this species was found, and many other apparently common *Bacteroides* species were not found, because only *B. caccae* represents the genus *Bacteroides* in the MG-RAST reference database. The BLAST algorithm behind MEGAN did use the same reference as Genometa, but uses an additional Lowest Common Ancestor (LCA) algorithm, which then apparently leads to marked differences in numbers of reads attributed. Use of the LCA algorithm reduces false positives, but has the undesirable effect of assigning many reads to high taxonomic ranks and discarding many others [30]. Genometa has the added advantage that users are able to check the distributions of reads across the genomes of each discovered species, in order to remove those with multiple hits to restricted genomic loci. These are likely to be false positives, for example due to the presence of homologous regions such as common genomic islands, or low quality reads aligning to low complexity genomic regions. As a consequence of observations made during the testing phase, we provide some recommendations for metagenome analysis algorithms on reads of different lengths (Table 2).

**Table 2:** Software recommendations for analysis of different types of metagenome datasets.

Metagenome dataset type	Read length	Recommended algorithms	Reference
16S rDNA	400	QIIME, Mothur, RDP classifier	[35], [36], [37]
Whole genome shotgun 454/Ion torrent	200–400	MEGAN/MG-RAST WebMGA/EBI/FR-HIT	[24], [25], [13], unpublished, [38]
Whole genome shotgun SOLiD/Illumina	50–120	Genometa/MG-RAST	This study, [25]

While Genometa was accurate on these datasets, it must be noted that the human associated microflora has been extremely well characterised by complete or draft genome sequences. Genometa can only attribute reads to those species with genomes contained within the reference sequence used. For this reason the program will do a good job on any habitats which have a thoroughly characterised microbiota in terms of reference genomes, but not perform well on samples from environments, such as soil, with a sparse coverage of fully sequenced or permanent draft genomes. However, this limitation is likely to become less prominent given that the current costs of whole genome draft bacterial sequencing are in the order of a few hundred euros. Complementary approaches such as de novo assembly can be used to discover truly novel genes or DNA fragments in environments where reference sequences are still lacking.

A further goal was to test the practicality of using current programs to assess the sizeable datasets generated by modern sequencing techniques. Genometa's run time is shorter than that of other approaches, with the Bowtie algorithm employed being orders of magnitude faster than BLAST (Table 1), and has no limits on the quantity of data which can be processed. Some web server based approaches, such as Webcarma3, restrict the amount of sequence data that can be uploaded in order to reduce run times, and other webservers use a queue system which may require some time before analyses actually run. For this reason only 100,000 reads from the Illumina human gut dataset were used, and not the millions of reads from the full dataset. This speed limitation is particularly relevant in a clinical environment, where rapid analysis is crucial [31]. On the other hand, webserver-based analyses frequently allow more comprehensive analyses than most standalone programs to date.

A key advantage of using data from whole genome sequencing metagenomic studies is to gain information on the gene content of the microbial community. That is, the coverage of genes by sequencing reads can infer the presence or absence of a gene or genomic island in the community (Materials S1, Table S1, Figures S1, S2). This approach potentially offers much more significant information than targeting the 16S rRNA gene alone [32]. The gene content of a taxon may have changed even though the rRNA sequence has not, especially through the integration of significant drivers of variation such as phages and genomic islands [33], [34]. Lastly, functional information can be used independently of taxonomy. Taxonomy can be arbitrary in some cases, but gene content is not.

As sequence datasets become ever more ubiquitous, we suggest a use case for programs like Genometa might be to easily check for contaminants in sequence datasets. Confounding sequences can be easily excluded following the alignment step. This could be human sequences in bacterial datasets, or eukaryotic sequences in bacterial datasets, or cloning vectors in both. We have already demonstrated the utility of this approach on several datasets which had acquired a minor amount of human contamination. Furthermore, alternative reference datasets can be rapidly built by our program RefSelector or concatenating genome sequences and used for custom alignments, for example when screening for rare virus subpopulations in a clinical metagenome.

## Conclusion

We here present a new graphical and user-friendly tool for analysis of short metagenomic sequence reads. Results obtained are similar to those from current metagenomic tools for existing datasets. By utilising algorithms orders of magnitude faster than Blast we aim to facilitate analysis of metagenomic short read data by workgroups which lack embedded bioinformaticians and computational infrastructure. We anticipate short reads will soon become more widely adopted in metagenomics, particularly for assessing functional information such as gene content.

## Supporting Information

Figure S1 Distribution of *Pseudomonas aeruginosa* CHA reads mapped to the *Herminiimonas arsenicoxydans* genome. Mapped reads correspond to ORFs from the RGP27 island in *P. aeruginosa* PACS2.
(TIF)

Figure S2 Distribution of human gut metagenome reads mapped to the *Bifidobacterium longum* NCC2705 genome. The widespread hits indicate the strain is present in the metagenome. Figures were produced with the statistical language R.
(TIF)

Materials S1 Supplementary results and statistical algorithms for short reads.
(DOC)

Table S1 P-values (i.e. probability) that not one single overlap is observed for any possible pair of reads among a particular number of mapped reads for a sequence of length L bp. This table was calculated from equation 5 above. Underlined p-values are those where at least one pair of overlapping reads are expected (p≲0.5) for the given sequence length and number of reads.
(DOC)
