# Supplementary figures and images for: Genometa - A Fast and Accurate Classifier for Short Metagenomic Shotgun Reads
Source: PLoS One. 2012 Aug 21;7(8):e41224. doi: 10.1371/journal.pone.0041224 (PMC3424124; doi:10.1371/journal.pone.0041224)

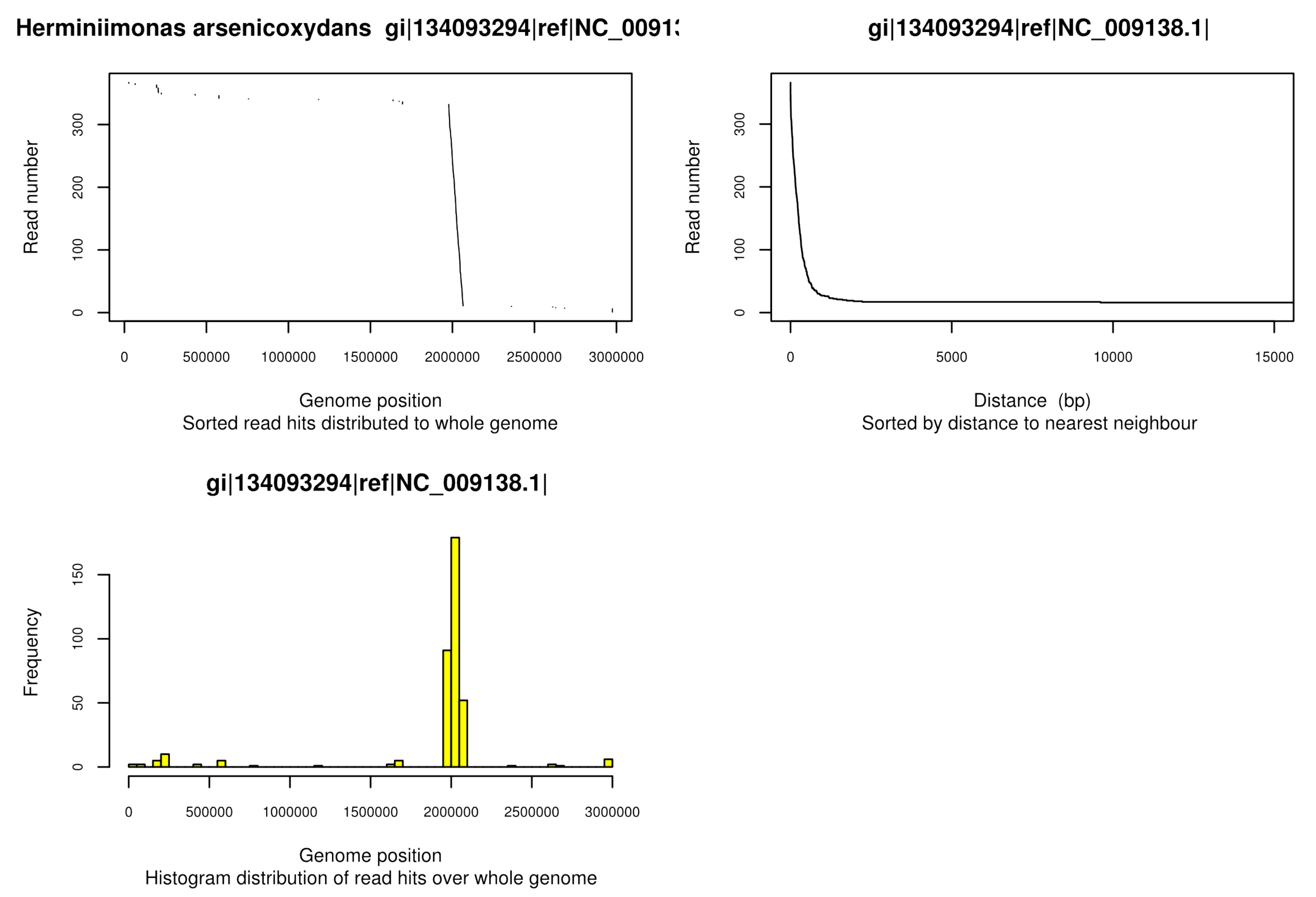

Supplement: Figure S1 — Distribution of Pseudomonas aeruginosa CHA reads mapped to the Herminiimonas arsenicoxydans genome. Mapped reads correspond to ORFs from the RGP27 island in P. aeruginosa PACS2. (TIF) [file pone.0041224.s001.tif]

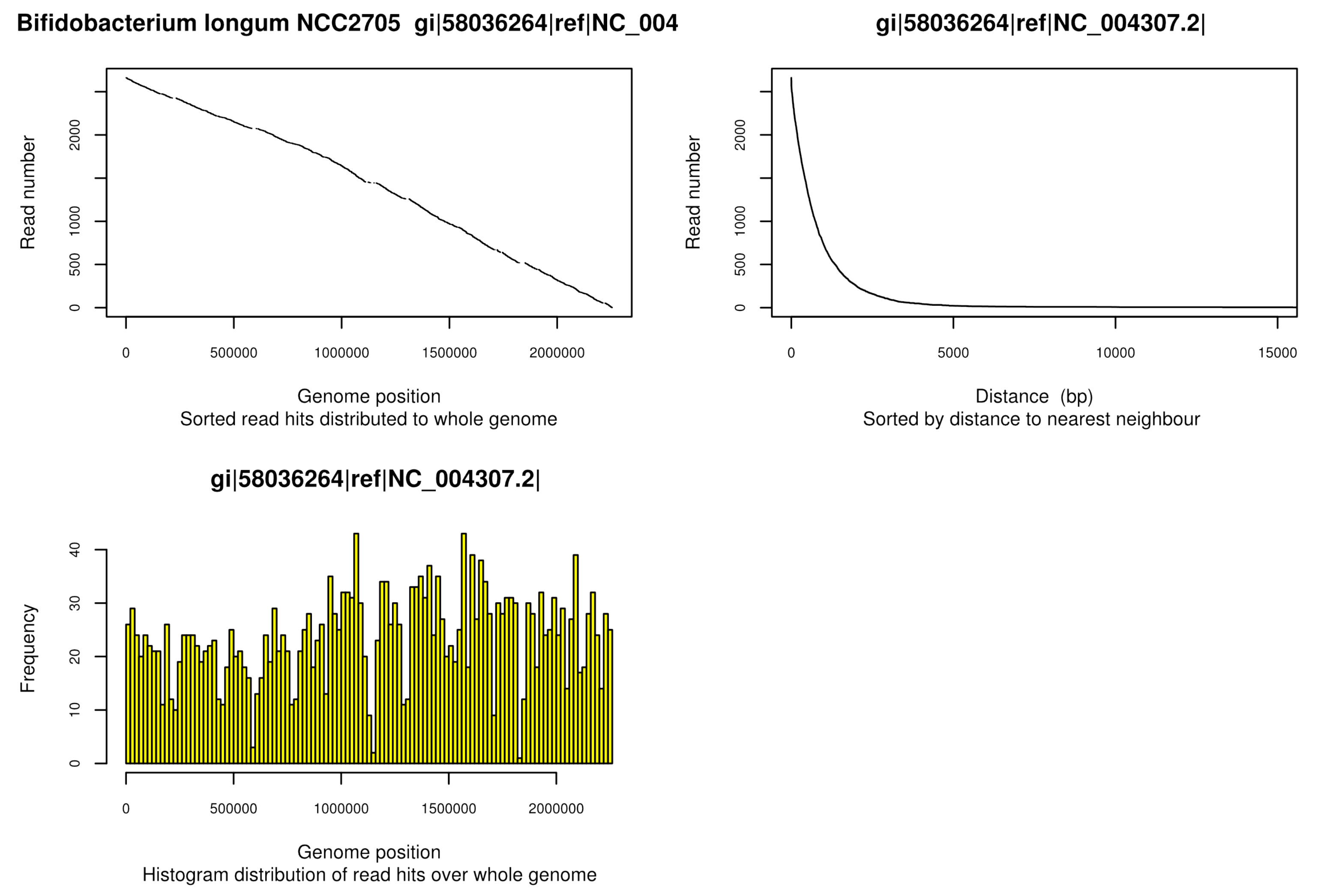

Supplement: Figure S2 — Distribution of human gut metagenome reads mapped to the Bifidobacterium longum NCC2705 genome. The widespread hits indicate the strain is present in the metagenome. Figures were produced with the statistical language R. (TIF) [file pone.0041224.s002.tif]
