# Supplementary material for: Genometa - A Fast and Accurate Classifier for Short Metagenomic Shotgun Reads
Source: PLoS One. 2012 Aug 21;7(8):e41224. doi: 10.1371/journal.pone.0041224 (PMC3424124; doi:10.1371/journal.pone.0041224)
Supplement: Materials S1 — Supplementary results and statistical algorithms for short reads. (DOC) [file pone.0041224.s003.doc]

**Supplementary Information**

**Davenport et al. Genometa - a fast and accurate classifier for short metagenomic shotgun reads**

**Supplementary Methods**

Reference Genome Dataset

Notation for unfinished (non-RefSeq) genomes

The usual RefSeq notation, e.g. NC_000001, was slightly modified to accomodate unfinished genomes which are not yet (and may never be) included in RefSeq.

NC_m00022 source: the Moore microbial sequencing project.

NC_i00019 source: human microbiome project, retrieved from Integrated Microbial Genomes.

NC_j00001 source: GEBA project, retrieved from the Joint Genome Institute.

NC_s00001 source: Metahit project, from the Sanger centre.

**Supplementary Results**

**Novel statistical algorithms for short reads**

**Algorithm 1: Assessment of the average distances of all reads from each other in a *circular* bacterial genome**

If$N$is the genome length, possible distances between two reads range between$0$and$\frac{N}{2}$bp. The average distance of two short reads in a bacterial genome is thus:

$$\bar{x}=\frac{N}{4}$$

where$N$is the genome length in nucleotides. If$n_{i}$is the number of reads, then

$\bar{x}\left(n_{i}\right)=\frac{N}{4}$(1)

In practice,$\bar{x}$is calculated as the average distance between *all pairs* of reads aligned to a genome. Genome size$N$can thus be estimated for circular genomes, given a sufficient number of reads$n_{i}$.

**Algorithm 2: Calculation of the average distance of two short sequence reads in a *linear* sequence**

In a genomic interval of length$L$one sequence read will match with$r$nucleotides of$L$.

$$N=L-r$$

where$N$is the number of nucleotides not occupied by one sequence read, and$r$is the read length in bp (r=100bp might be typical for a Illumina HiSeq read).

The total number of possibilities$A$of two reads $r_{1}$and$r_{2}$ of identical length$r$which are within$x_{i}$nucleotides from each other, $0\leq x_{i}\leq N$is

$$A=\sum_{i=0}^{N} x_{i}=N\frac{\left(N+1\right)}{2}$$

At the average distance$\bar{x}$the number of possibilities varies between$N$for $x=0$(two matching reads) and$\left(N-\bar{x}\right)()$for $x= \bar{x}$.

The total number of possibilities within the distances 0 and$\bar{x}$is

$$\bar{x} ^{2}-\left(\text{2N}-1\right)\cdot\bar{x}+N\frac{\left(N+1\right)}{2}-2N=0$$

(2)

For large$N$($N$> 100), as is typical for a sequence representing at least one gene,$\bar{x}$is approximated by

$\bar{x}=\left(1-\frac{1}{2}\sqrt{2}\right)N$(2')

Equations 1, 2, and 2' can be used to estimate the length $L\approx N$of the sequence that is covered by short sequence reads.

For the case of a circular sequence this is

$N=4\bar{x}\cdot b$(3)

and for the case of a linear sequence this is

(4)

Assuming that the$n_{i}$sequence reads randomly cover the target sequence$L$, the 95% and 99% confidence intervals for$N$are given by

$$b=\frac{\left(n_{i}\right)}{\left(n_{i}\pm a\sqrt{n_{i}}\right)}$$

where$a$= 1.96 (p < 0.05) and$a$= 2.57 (p < 0.01) respectively.

Equations 3 and 4 provide information from the experimentally derived sequence reads of whether the reads map

- to the whole chromosome of a taxon which is represented by at least one sequenced genome in the database or
- to the chromosome of a taxon that is closely related to at least one sequenced strain

or whether the reads map

- onto a gene, operon, genomic island etc of which homologous sequence is present in completely sequenced genomes.

In other words, equations 3 and 4 allow predictions about the relative abundance of taxa with completely sequenced reference genomes and of known genes, operons and genomic islands in yet unknown taxa.

**Example 1:** A genomic island from *Herminiimonas arsenicoxydans* is present in the *P. aeruginosa* CHA reads according to Supplementary Figure S1. As this is a linear sequence we can use equation 4 to estimate the size of this island.

We first reduce the data to that around the genomic island (within the interquartile range of the dataset). After empirically calculating the average distance$\bar{x}$with an in-house R script, we can then substitute the value 17101 bp into equation 4. $N$is then estimated as 58387 bp (95% confidence intervals 51033, 68216). The actual genomic island in question is related to the RGP27 island in *P. aeruginosa* PACS2 which has an actual size of circa 80000 bp.

**Example 2:** The whole genome of *Bifidobacterium longum* NCC2705 appears to be present in the human gut metagenome dataset according to Supplementary Figure S2. We can estimate genome size mathematically by using equation 3.

The average distance$\bar{x}$ can again be empirically calculated (=738557 bp). Using equation 3, we then get an estimated genome size of 2954229 bp (95% confidence intervals 2846109, 3070887). The estimate of 2.9 Mb is significantly larger than the true *B. longum* genome size of 2.25 Mb. This indicates that foreign DNA from related but unsequenced strains is present in the metagenome, which also maps to the *B. longum* genome. This phenomenon disturbs the assumption of random distribution of reads.

**Algorithm 3: Probability that no overlaps between reads occur**

Complementary to equations 3 and 4, we calculate the probability that in a sequence of length $L$none of $n_{i}$ sequence reads overlap with each other by 11 or more base pairs. In case of an Illumina read of 36 bp, the probability$\lambda$for an overlap is

$$\lambda=\frac{\left(52\right)}{L}$$

and the probability$P\left(O\right)$that not one single overlap is observed for any possible pair among $n_{i}$reads becomes

$P\left(O_{\left(n_{i}\right)}\right)=\left(\left(1\right)-\lambda\right)^{\left(n_{i}\frac{\left(n_{i}-\left(1\right)\right)}{\left(2\right)}\right)}$(5)

Equation 5 again allows discrimination of whether the reads that map onto the concatenated sequence pangenome map onto a gene $\left(10^{2}<L<10^{4}\right)$, an operon $\left(10^{3}<L<10^{4}\right)$, a genomic island $\left(10^{4}<L<10^{5}\right)$, or onto a whole chromosome $\left(10^{6}<L<10^{7}\right)$. In a sequence of length$10^{2} ,10^{4} ,10^{5} ,10^{6}$bp one can see on average at least one overlap with 5, 10-20, 40-60, or 100-200 reads respectively (see Supplementary Table S1 below for sample values).

In summary, these algorithms add value to the short read metagenomic approach demonstrated by Genometa and allow further predictions and characterisation of taxa which have been predicted to be present by the original analysis steps.

**Recommended Bowtie alignment settings for longer or paired end reads**

Bowtie is capable of aligning reads up to 100bp, about the longest currently attainable with the Illumina instrument, and beyond. For reads 50bp or longer, users are advised to increase the alignment seed length using the "-l" option. It is also advisable to increase the sensitivity of the alignment policy by increasing the quality-distance ceiling with the "-e" option. For 50bp reads, reasonable options are "-n 3 -l 30 -e 140." For 75bp reads, reasonable options are "-n 3 -l 40 -e 200". Depending on the quality of the reads, users may also benefit from setting trimming thresholds using Bowtie's "-3" and "-5" options. Users are encouraged to experiment with different parameters to determine which yield the best combination of speed and sensitivity.

Bowtie is also capable of aligning paired-end reads using the "-1" and "-2" options. For paired-end alignment, it is critical that the "-I" and "-X" parameters be set to reflect the expected range of insert lengths, and that the "--ff/--fr/--rf" option be set to correctly reflect the expected relative orientation of the mates. Otherwise, the alignment policy should be set similarly to the unpaired case.

For further details, see the Bowtie manual.

**References**

Richter DC, Ott F, Auch AF, Schmid, R, Huson DH. MetaSim: a sequencing simulator for genomics and metagenomics. PLoS ONE 2008, 3: e3373.
