# Supplementary material for: Genometa - A Fast and Accurate Classifier for Short Metagenomic Shotgun Reads
Source: PLoS One. 2012 Aug 21;7(8):e41224. doi: 10.1371/journal.pone.0041224 (PMC3424124; doi:10.1371/journal.pone.0041224)
Supplement: Table S1 — P-values (i.e. probability) that not one single overlap is observed for any possible pair of reads among a particular number of mapped reads for a sequence of length L bp. This table was calculated from equation 5 above. Underlined p-values are those where at least one pair of overlapping reads are expected (p≲0.5) for the given sequence length and number of reads. (DOC) [file pone.0041224.s004.doc]

**Supplementary Table S1** P-values (i.e. probability) that not one single overlap is observed for any possible pair of reads among a particular number of mapped reads for a sequence of length L bp. This table was calculated from equation 5 above. Underlined p-values are those where at least one pair of overlapping reads are expected (p≲0.5) for the given sequence length and number of reads.

| Number of reads | | | | | | | | |
| --- | --- | --- | --- | --- | --- | --- | --- | --- |
| L (bp) | 2 | 5 | 10 | 20 | 50 | 100 | 200 | 500 |
| 10^2^ | 0.23 | <0.001 |  |  |  |  |  |  |
| 10^3^ | 0.95 | 0.59 | 0.09 | <0.001 |  |  |  |  |
| 10^4^ | 0.99 | 0.95 | 0.79 | 0.37 | <0.01 | <0.001 |  |  |
| 10^5^ |  | 0.99 | 0.98 | 0.91 | 0.53 | 0.08 | <0.001 |  |
| 10^6^ |  |  |  | 0.99 | 0.94 | 0.77 | 0.36 | <0.01 |
